# Pangenome comparison via ED strings

**DOI:** 10.3389/fbinf.2024.1397036

**Published:** 2024-09-26

**Authors:** Esteban Gabory, Moses Njagi Mwaniki, Nadia Pisanti, Solon P. Pissis, Jakub Radoszewski, Michelle Sweering, Wiktor Zuba

**Affiliations:** ^1^ Centrum Wiskunde & Informatica, Amsterdam, Netherlands; ^2^ Department of Computer Science, University of Pisa, Pisa, Italy; ^3^ Department of Computer Science, Vrije Universiteit, Amsterdam, Netherlands; ^4^ Institute of Informatics, University of Warsaw, Warsaw, Poland

**Keywords:** elastic-degenerate string, intersection graph, pangenome comparison, matching statistics, SARS-CoV-2

## Abstract

**Introduction:**

An elastic-degenerate (ED) string is a sequence of sets of strings. It can also be seen as a directed acyclic graph whose edges are labeled by strings. The notion of ED strings was introduced as a simple alternative to variation and sequence graphs for representing a pangenome, that is, a collection of genomic sequences to be analyzed jointly or to be used as a reference.

**Methods:**

In this study, we define notions of *matching statistics* of two ED strings as similarity measures between pangenomes and, consequently infer a corresponding distance measure. We then show that both measures can be computed efficiently, in both theory and practice, by employing the *intersection graph* of two ED strings.

**Results:**

We also implemented our methods as a software tool for pangenome comparison and evaluated their efficiency and effectiveness using both synthetic and real datasets.

**Discussion:**

As for efficiency, we compare the runtime of the intersection graph method against the classic product automaton construction showing that the intersection graph is faster by up to one order of magnitude. For showing effectiveness, we used real SARS-CoV-2 datasets and our matching statistics similarity measure to reproduce a well-established clade classification of SARS-CoV-2, thus demonstrating that the classification obtained by our method is in accordance with the existing one.

## 1 Introduction

Many biomedical applications of bioinformatics face the twofold challenge of analyzing an ever-increasing number of genome sequences and the need to choose which genome should be used as the *reference*. Generalizing other definitions, in The computational Pangenomics Consortium (2018), a *pangenome* was defined as “any collection of genomic sequences to be analyzed jointly or to be used as a reference,” somewhat merging the two above-mentioned challenges into that of analyzing a pangenome. When projected within a single species, a pangenome represents a collection of sequences that are (part of whole) genomes originating from different individuals or strains of a single clade or population.

Currently, *pangenomics* constitutes an important paradigm shift within genomics to deal with the widespread availability of human sequencing data and the discovery of large-scale genomic variation in many eukaryotic species; see Paten et al. (2017); Liao et al. (2023). In contrast to a *linear* reference, a pangenome reference aims to compactly represent the variation within a population by encoding the commonalities and differences among the underlying sequences. This gives rise to different pangenome representations, usually edge- or node-labeled directed graphs (Baaijens et al., 2022; Carletti et al., 2019). The most widely-used pangenome representations are *variation graphs* (see Garrison et al., 2018a; Eizenga et al., 2021) and *sequence graphs* (see Rakocevic et al., 2019).

The computational challenges posed by pangenomes often result in a trade-off between the efficiency and accuracy of the methods and the information content of the chosen representation. A simpler *acyclic* alternative to the aforementioned representations is the notion of *elastic-degenerate string* (ED string); see Iliopoulos et al. (2021). When compared to more powerful representations, ED strings have the algorithmic advantage of supporting both theoretically (Aoyama et al., 2018; Bernardini et al., 2019; 2022)) and practically (Grossi et al., 2017; Pissis and Retha, 2018; Cisłak et al., 2018) fast *on-line* pattern matching, also for the approximate case (Bernardini et al., 2017; 2020). Moreover, they support fast dynamic-programming-based alignment, as shown in Mwaniki et al. (2023); Mwaniki and Pisanti (2022), and short-read mapping; see Büchler et al. (2023).

**FIGURE 1:**
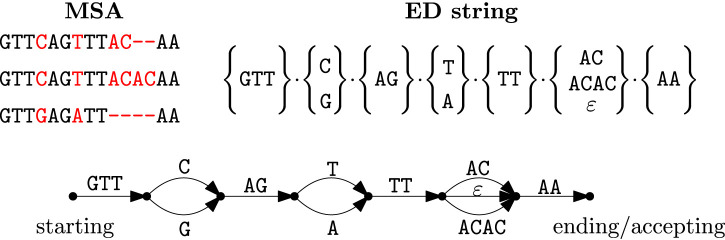
An example of an MSA (top left) and its corresponding (non-unique) ED string T of length n=7 , cardinality m=11 and size N=20 (top right), and edge-labeled DAG for T . Note that ε denotes the empty string. The DAG can also be viewed as an NFA with extended (multi-letter) transitions.

An ED string is a concatenation of n sets of strings (inspect Figure 1). Every set of strings encodes a collection of *consecutive columns* of an underlying multiple sequence alignment (MSA), from left to right. Every set encodes the commonalities or differences of the underlying sequences which are represented by the MSA. Formally, an ED string T is a sequence of n sets T[1],…,T[n] containing m strings in total whose cumulative length is N . We call n , m , and N the *length*, the *cardinality* and the *size* of T , respectively. An ED string T compactly represents all strings that can be spelled concatenating, for 1≤i≤n , a string chosen from set T[i] . For example, the string GTTCAGATTACAA is one of the strings represented by the ED string of Figure 1. Every ED string can also be viewed as an edge-labeled directed acyclic graph (DAG). As an example, Figure 1 shows the DAG of a simple ED string: the DAG has n+1 nodes as the ED string has length n=7 . The ED string may also be viewed as a nondeterministic finite automaton (NFA) Hopcroft et al. (2003) with extended transitions. By *extended*, we mean multi-letter transitions, instead of single-letter ones.

Any pangenome representation aims to improve the downstream analyses of genomic data by removing biases inherent in the use of a linear single-genome representation (Baaijens et al., 2022). For example, pangenomes allow for representing haplotype-resolved data with genome phasing, as shown in Bonizzoni et al. (2016). Using linear genomes as a reference, determining at which chromosomal copy (i.e., paternal or maternal) the different alleles are located, may be erroneous or incomplete due to reference bias. Genotyping (the task of reconstructing the allele variants that characterize an individual) can be improved by using a pangenome representation as a reference, which removes the bias of using a single linear genome as a reference to map the reads coming from an individual’s sample. Pangenomes also allow for accurate read alignment as certain genome regions are important yet challenging to assemble using classic read alignment tools, because of the bias of using a single linear reference genome (Garrison et al., 2018b; Liao et al., 2023). This explains the high level of attention paid in recent years to the task of sequence-to-graph comparison; see, e.g., Büchler et al. (2023); Equi et al. (2023); Mwaniki et al. (2023); Li et al. (2020); Gibney et al. (2022); Jain et al. (2020); Rautiainen et al. (2019); Rautiainen and Marschal (2020). In phylogenetic analyses, the aim is to study the evolutionary relationships among different groups of organisms (e.g., species or population variants). This was traditionally performed using a sample sequence per organism that is somewhat representative of the group or population, and then inferring a tree or a graph based on pairwise distances or similarities among these samples. This task can be biased if the sample linear genome turns out not to be the most representative, whereas a pangenome can compactly represent the entire population.

Our Contributions. Here, we make an important step from the above-mentioned *sequence-to-graph* (i.e., sequence-to-pangenome) comparison towards graph-to-graph (pangenome-to-pangenome) comparison^1^. In particular, we assume that the two pangenomes to be compared are represented by means of two ED strings: $T_{1}$ and $T_{2}$ . We first recall a very fast and low memory consumption method from Gabory et al. (2023) to determine whether $T_{1}$ and $T_{2}$ have a nonempty intersection, that is, whether the two pangenomes share a genome. This method relies on a powerful representation of all string fragments that are *in both* $T_{1}$ *and* $T_{2}$ , that is, of the complete set of sequences shared by the two pangenomes; this representation was named by Gabory et al. (2023) an *intersection graph* of $T_{1}$ and $T_{2}$ . From thereon, we develop a novel method for pangenome comparison via ED strings, based on an extension to ED strings of the well-known notion of Matching Statistics on standard strings (*cf.* the book Gusfield (1997)). We define two versions of Matching Statistics for ED strings.

• **ED Matching Statistics** as the theoretically most straightforward notion that extends the standard one: for every $i\in \left[1,n_{1}\right]$ of $T_{1}$ , where $n_{1}$ is the length of $T_{1}$ , we report in ${\text{MS}}_{T_{1},T_{2}}\left[i\right]$ the length of the longest string starting at the i th set of $T_{1}$ that is also a substring of $T_{2}$ .

• **Breakpoint Matching Statistics** as a notion specifically designed for genomic variants: for every $i\in \left[1,n_{1}\right]$ of $T_{1}$ , we report in ${\text{BMS}}_{T_{1},T_{2}}\left[i\right]$ the length of the longest string starting at the i th set of $T_{1}$ that is also a substring of $T_{2}$ and that is within pairs of breakpoints that have been detected by the multiple alignment underlying the pangenome.

The output is the Matching Statistics array ${\text{MS}}_{T_{1},T_{2}}$ (resp. ${\text{BMS}}_{T_{1},T_{2}}$ ) of size $n_{1}$ that specifies maximal local matches of $T_{1}$ with respect to $T_{2}$ . The Matching Statistics array ${\text{MS}}_{T_{2},T_{1}}$ (resp. ${\text{BMS}}_{T_{2},T_{1}}$ ) of $T_{2}$ with respect to $T_{1}$ is defined dually: for every $j\in \left[1,n_{2}\right]$ of $T_{2}$ , where $n_{2}$ is the length of $T_{2}$ , we report the longest string starting at the j th set of $T_{2}$ that fulfills the corresponding requirements with a substring of $T_{1}$ . We then suggest to use the Matching Statistics to define the following measures.

• Similarity measure $MS\left(T_{1},T_{2}\right)$ (resp. $BMS\left(T_{1},T_{2}\right)$ between $T_{1}$ and $T_{2}$ as the sum of the average values of arrays ${\text{MS}}_{T_{1},T_{2}}$ and ${\text{MS}}_{T_{2},T_{1}}$ (resp. of arrays ${\text{BMS}}_{T_{1},T_{2}}$ and ${\text{BMS}}_{T_{2},T_{1}}$ );

• Distance measure d (resp. bd ) between $T_{1}$ and $T_{2}$ based on $MS\left(T_{1},T_{2}\right)$ (resp. on $BMS\left(T_{1},T_{2}\right)$ ).

Both distance measures can be trivially computed in O(1) time from $MS\left(T_{1},T_{2}\right)$ and $BMS\left(T_{1},T_{2}\right)$ , and both similarity measures can be trivially computed in $O\left(n_{1}+n_{2}\right)$ time from ${\text{MS}}_{T_{1},T_{2}}$ and ${\text{MS}}_{T_{2},T_{1}}$ (resp. ${\text{BMS}}_{T_{1},T_{2}}$ and ${\text{BMS}}_{T_{2},T_{1}}$ ). The algorithmic challenge is thus: *how can we efficiently compute the Matching Statistics arrays*? While an algorithm based on classic product automaton techniques (Lawson, 2004) would require $\Omega \left(N_{1}N_{2}\right)$ time in the worst case, our method achieves this in $O\left(N_{1}m_{2}+N_{2}m_{1}\right)$ worst-case time, where $N_{1}$ and $N_{2}$ are the sizes of $T_{1}$ and $T_{2}$ , respectively, and $m_{1}$ and $m_{2}$ are the cardinalities of $T_{1}$ and $T_{2}$ , respectively. We achieve this via the above-mentioned intersection graph of $T_{1}$ and $T_{2}$ . The running time of our algorithm is good in the following sense: if each of the ED strings, $T_{1}$ and $T_{2}$ , represents a pangenome of closely-related genomes, then the cardinalities $m_{1}$ and $m_{2}$ are expected to be asymptotically much smaller than the sizes $N_{1}$ and $N_{2}$ , respectively.

We also implemented the entire pipeline in C++ as a software tool for pangenome comparison, which is publicly available at https://github.com/urbanslug/junctions under GNU GPL v3.0.

We evaluated the efficiency and effectiveness of the developed methods using both synthetic and real datasets. For efficiency, we compared the runtime of the intersection graph against the classic product automaton construction. As expected, the intersection graph is faster by up to one order of magnitude. For effectiveness, we used real SARS-CoV-2 datasets and our *Breakpoint Matching Statistics* method to reproduce a well-established clades classification of SARS-CoV-2, thus demonstrating that the classification obtained by our method is in accordance with the existing classification.

In Section 2, we describe and analyze our methods. In Section 3, we present our results. We conclude this paper in Section 4.

## 2 Methods

In this section we recall from Gabory et al. (2023) the ED String Intersection problem for two ED strings (Section 2.1) and the notion of *intersection graph* (Section 2.2). We then extend the Matching Statistics problem on two standard strings (Gusfield, 1997) to two ED strings defining the ED Matching Statistics and Breakpoint Matching Statistics problems, and show how to solve them using an intersection graph (Section 2.3). We also formally define our similarity and distance measures for ED strings (Section 2.4).

Let us begin with some basic definitions and notations from Gabory et al. (2023). An *alphabet* Σ is a finite nonempty set of elements called *letters*. By $\Sigma^{*}$ we denote the set of all strings over Σ including the *empty string* ε of length 0. An *elastic-degenerate string* (ED string, in short) T is a sequence T=T[1]⋯T[n] of n finite sets, where T[i] is a subset of $\Sigma^{*}$ . The total size of T is defined as $N=N_{\varepsilon }+\sum_{i=1}^{n}\sum_{S\in T\left[i\right]}|S|$ , where $N_{\varepsilon }$ is the total number of empty strings in T . By m we denote the total number of strings in all T[i] , i.e., $m=\sum_{i=1}^{n}|T\left[i\right]|$ . We say that T has *length* n=|T| , *cardinality* m and *size* N . The *language* of T is defined as $L\left(T\right)=\left\{S_{1}\cdot \ldots \cdot S_{n}\;:\;S_{i}\in T\left[i\right]\text{ for all }i\in \left[1,n\right]\right\}$ , where ⋅ denotes the string concatenation.

### 2.1 ED strings intersection

Let us start by formally defining the ED String Intersection problem.

ED String Intersection (EDSI)

Input: Two ED strings, $T_{1}$ of length $n_{1}$ , cardinality $m_{1}$ and size $N_{1}$ , and $T_{2}$ of length $n_{2}$ , cardinality $m_{2}$ and size $N_{2}$ .

Output: YES if $L\left(T_{1}\right)$ and $L\left(T_{2}\right)$ have a nonempty intersection, NO otherwise.

This EDSI problem can be efficiently solved using the notion of *compacted nondeterministic finite automaton* (compacted *NFA*). A compacted NFA is a 5-tuple $\left(Q,\Sigma ,\delta^{\mathrm{ext}},q_{0},F\right)$ , where Q is a finite set of *states*; Σ is an alphabet; $\delta^{\mathrm{ext}}:Q\times \Sigma^{*}\to P\left(Q\right)$ , is an *extended transition* function, P(Q) is the power set of Q ; $q_{0}\in Q$ is the *starting* state; and F⊆Q is the set of *accepting* states. Such an NFA can also be represented by a standard (uncompacted) NFA, where each extended transition is subdivided into standard one-letter transitions (and ε -transitions), δ:Q×(Σ∪{ε})→P(Q) . The states of a compacted NFA are called *explicit*, whereas the states obtained from these subdivisions are called *implicit*. In both cases, given an (compacted or not) NFA A , we define the *language accepted by* A , denoted by L(A) , as the set of strings that can be read from the starting state to an accepting state.

We next define the path-automaton of an ED string.

Definition 1 (Path-automaton of an ED string). *Let* T *be an ED string of length* n *, cardinality* m *, and size* N *. The path-automaton of* T *is the compacted NFA consisting of* V *states and* E *transitions defined as follows:*

• V=n+1 *is the number of explicit states, numbered from one through* n+1 *. State one is the starting state and state* n+1 *is the accepting state. State* i∈[2,n] *is the state in-between* T[i−1] *and* T[i] .

• $E=m=\sum_{i}m_{i}$ *, where* $m_{i}=|T\left[i\right]|$ *is the number of extended transitions from state* i *to state* i+1 *labeled with the strings in* T[i] .

*The path-automaton of* T *accepts exactly* L(T) *. The uncompacted version of this path-automaton has* $V^{u}=O\left(N\right)$ *states and* $E^{u}=N$ *transitions.*

In the following, we are interested only in the graph underlying the path-automaton, that is, the directed acyclic graph (DAG), where every *node* represents an explicit state and every labeled directed *edge* represents an extended transition of the path-automaton (inspect also Figure 1). Indeed, it should be noticed that the path-automata of the ED strings are DAGs (direct acyclic graphs) as they are always acyclic, but may contain ε -transitions. For example, the DAG shown in Figure 1 is the path-automaton for T .

Checking whether two NFA have a nonempty intersection can be done in $O\left(N_{1}N_{2}\right)$ time^2^,^3^, where $N_{1}$ and $N_{2}$ are the sizes of the two NFA, and therefore a naïve method can check whether $L\left(T_{1}\right)\cap L\left(T_{2}\right)\neq \emptyset$ in time $O\left(N_{1}N_{2}\right)$ , that is, quadratic in the sizes of $T_{1}$ and $T_{2}$ . The compacted NFA representation allows for a more efficient algorithm for computing and representing the intersection. The idea is to use compacted transitions that directly compare whole string-segments instead of single letters, which can be performed efficiently using classic tools such as suffix trees or LCP queries.

Lemma 1 Gabory et al. (2023). Given two compacted NFA $A_{1}$ and $A_{2}$ , with $V_{1}$ and $V_{2}$ explicit states and $E_{1}$ and $E_{2}$ extended transitions, respectively, a compacted NFA representing the intersection of $A_{1}$ and $A_{2}$ with $O\left(V_{1}^{u}V_{2}+V_{1}V_{2}^{u}\right)$ explicit states and $O\left(E_{1}^{u}E_{2}+E_{1}E_{2}^{u}\right)$ extended transitions can be computed in $O\left(E_{1}^{u}E_{2}+E_{1}E_{2}^{u}\right)$ time.

Lemma 1 thus implies the following result.

Corollary 1 The compacted NFA representing the intersection of two path-automata with $O\left(N_{1}n_{2}+N_{2}n_{1}\right)$ explicit states and $O\left(N_{1}m_{2}+N_{2}m_{1}\right)$ extended transitions can be constructed in $O\left(N_{1}m_{2}+N_{2}m_{1}\right)$ time.

Consequently, we can compute the compacted NFA of the intersection of two ED strings $T_{1}$ and $T_{2}$ (with cardinalities $m_{1}$ and $m_{2}$ and sizes $N_{1}$ and $N_{2}$ ) in $O\left(N_{1}m_{2}+N_{2}m_{1}\right)$ time. We remark that this compacted NFA is also an acyclic graph which we name the *intersection graph* of the two ED strings. As shown by Gabory et al. (2023), one can solve EDSI in practice without effectively constructing the entire graph, but rather part of it. However, since the intersection graph is crucial for the other methods that we describe in Section 2.3 and apply in this paper, we dedicate the following section to its description.

### 2.2 The intersection graph

In this section we describe the notion of the *intersection graph* G from the DAGs (of the two path-automata) $G_{1}$ and $G_{2}$ of $T_{1}$ and $T_{2}$ and how it can be used to solve the EDSI problem. We will do this by means of a running example of two ED strings $T_{1}$ and $T_{2}$ . We refer the reader to Gabory et al. (2023) for the formal definition of G and the full details of an efficient $O\left(N_{1}m_{2}+N_{2}m_{1}\right)$ -time construction algorithm.

**FIGURE 2:**
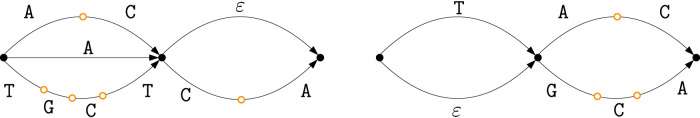
The two DAGs $G_{1}$ and $G_{2}$ for ED strings $T_{1}$ and $T_{2}$ . The filled black nodes are explicit states, while the orange empty nodes are implicit states.

Let us consider the two ED strings $T_{1}=\left\{\begin{array}{c} AC\\ A\\ TGCT \end{array}\right\}\cdot \left\{\begin{array}{c} \varepsilon \\ CA \end{array}\right\}$ and $T_{2}=\left\{\begin{array}{c} T\\ \varepsilon \end{array}\right\}\cdot \left\{\begin{array}{c} AC\\ GCA \end{array}\right\}$ that have a nonempty intersection as the string AC belongs to $L\left(T_{1}\right)\cap L\left(T_{2}\right)$ . Their path-automata are represented by graphs $G_{1}$ and $G_{2}$ shown in Figure 2.

**FIGURE 3:**
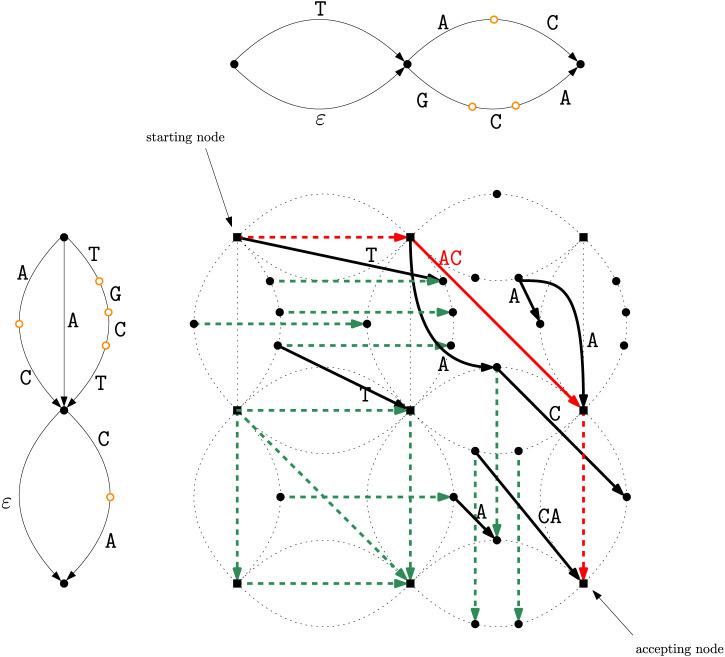
Intersection graph G for $T_{1}$ and $T_{2}$ , where $G_{1}$ and $G_{2}$ are shown at the left and on the top, respectively, to simplify the understanding of G . A node (i,j) in the intersection is represented by a square if both i and j are explicit nodes, and by a circle if only one of them is. The dashed edges of the intersection graph G correspond to ε -transitions (namely, transitions such that no letter is read when traversed), while the solid edges correspond to the other extended transitions. A string in $L\left(T_{1}\right)\cap L\left(T_{2}\right)$ corresponds to a path from the starting node of G to the accepting node. Here the intersection is nonempty and contains a single string AC , which can be read on the red path.

The nodes of the intersection graph correspond to pairs (i,j) from $G_{1}$ and $G_{2}$ , respectively, where at least one of them must be an explicit state. As a consequence, we draw the intersection graph using, as a layout, a grid (in dotted lines) of $n_{2}+1$ copies of $G_{1}$ and $n_{1}+1$ copies of $G_{2}$ . Therein, the possible nodes for the intersection graph G are pairs, as described above. Let (i,j) and $\left(i^{\prime },j^{\prime }\right)$ be nodes in G , with $i,i^{\prime }$ from $G_{1}$ and $j,j^{\prime }$ from $G_{2}$ . We observe an extended transition from (i,j) to $\left(i^{\prime },j^{\prime }\right)$ with label S if one can read a string S both from i to $i^{\prime }$ in $G_{1}$ and from j to $j^{\prime }$ in $G_{2}$ . Figure 3 shows the intersection graph G for $T_{1}$ and $T_{2}$ : the intersection is nonempty and contains a single string AC that can be read on the red path.

### 2.3 Matching statistics for ED strings

When the ED strings $T_{1}$ and $T_{2}$ come from real pangenomes, being able to quickly tell whether $L\left(T_{1}\right)\cap L\left(T_{2}\right)$ is nonempty might not be informative enough for practical applications. Indeed, two pangenomes may still share a lot of fragments even if the two ED strings that represent them are such that $L\left(T_{1}\right)\cap L\left(T_{2}\right)=\emptyset$ . Thus, to be more sensitive to local similarities and detect shared *fragments* of pangenomes, we consider *Matching Statistics* (Gusfield, 1997), a more elaborate ED string comparison task that is heavily employed for standard strings (under the Hamming distance model, i.e., with mismatches) in practical applications, especially in bioinformatics (Ulitsky et al., 2006; Apostolico et al., 2014; Leimeister and Morgenstern, 2014; Apostolico et al., 2016; Pizzi, 2016; Thankachan et al., 2017).

Let us first recall the classic Matching Statistics problem on standard strings.

Matching Statistics

Input: Two strings, $S_{1}$ of length $n_{1}$ , and $S_{2}$ of length $n_{2}$ . 

Output: For each $i\in \left[1,n_{1}\right]$ , the length ${\text{MS}}_{S_{1},S_{2}}\left[i\right]$ of the longest prefix of $S_{1}\left[i.\;.n_{1}\right]$ , which is a substring of $S_{2}$ .

For example, the Matching Statistics of $S_{1}=AGTGCATTG$ of length nine and $S_{2}=TTG$ are the following array of size $|S_{1}|=9$ : ${\text{MS}}_{S_{1},S_{2}}=\left[0,1,2,1,0,0,3,2,1\right]$ . The Matching Statistics problem can be solved in linear $O\left(n_{1}+n_{2}\right)$ time using the suffix tree of $S_{1}$ (Gusfield, 1997). In this section we extend the Matching Statistics notion, as well as the problem of computing them, to ED strings, in the direction of representing local similarities for pangenomes. We suggest two possible definitions of Matching Statistics for ED strings: the first one (Section 2.3.1) is the most inclusive notion, that is, it takes into account local matches that are prefixes of a string in $L\left(T_{1}\left[i.\;.n_{1}\right]\right)$ and occur in some string from $L\left(T_{2}\right)$ ; the second notion (Section 2.3.2) has a biologically motivated further condition: it assumes that relevant fragments are those that begin at positions of the genomes that the multiple alignment has detected as a *breakpoint*, meaning a locus of the resulting pangenome in which a variant (or a conserved fragment) either begins or ends. We will name the former notion *ED Matching Statistics* and the latter *Breakpoint Matching Statistics*.

#### 2.3.1 ED matching statistics

The *ED Matching Statistics* between two ED strings $T_{1}$ of length $n_{1}$ and $T_{2}$ of length $n_{2}$ , is an array ${\text{MS}}_{T_{1},T_{2}}$ of length $n_{1}$ storing, for each $i\in \left[1,n_{1}\right]$ , the length of the longest local match between $T_{1}$ and $T_{2}$ which is a prefix of a string in $L\left(T_{1}\left[i.\;.n_{1}\right]\right)$ .

ED Matching Statistics

Input: Two ED strings, $T_{1}$ of length $n_{1}$ , cardinality $m_{1}$ and size $N_{1}$ , and $T_{2}$ of length $n_{2}$ , cardinality $m_{2}$ and size $N_{2}$ . 

Output: For each $i\in \left[1,n_{1}\right]$ , the length ${\text{MS}}_{T_{1},T_{2}}\left[i\right]$ of the longest prefix of a string in $L\left(T_{1}\left[i.\;.n_{1}\right]\right)$ , which is a substring of a string in $L\left(T_{2}\right)$ .

Example 1 Let us consider again $T_{1}=\left\{\begin{array}{c} AC\\ A\\ TGCT \end{array}\right\}\cdot \left\{\begin{array}{c} \varepsilon \\ CA \end{array}\right\}$ and $T_{2}=\left\{\begin{array}{c} T\\ \varepsilon \end{array}\right\}\cdot \left\{\begin{array}{c} AC\\ GCA \end{array}\right\}$ of our running example. We have that ${\text{MS}}_{T_{1},T_{2}}\left[1\right]=3$ and ${\text{MS}}_{T_{1},T_{2}}\left[2\right]=2$ . Indeed, the longest prefix of a string in $L\left(T_{1}\right)$ that occurs in $L\left(T_{2}\right)$ is TGC , having length 3, and the longest prefix of a string in $L\left(T_{1}\left[2\right]\right)$ that occurs in $L\left(T_{2}\right)$ is CA , having length 2.

We observe that, in the intersection graph G of $T_{1}$ and $T_{2}$ , the sought match starts at a node (i,j) where i is an explicit state of $T_{1}$ . As a consequence, the intersection graph G can be used to efficiently compute the Matching Statistics of two ED strings, using the following algorithm:

We consider a slightly augmented version of the intersection graph obtained from an uncompacted intersection automaton. We first construct the automaton as in Corollary 1, and then we additionally compute some extra nodes and transitions as follows: when we process a state corresponding to a pair (i,j) (where i is from $G_{1}$ and j from $G_{2}$ ), and we have two transitions s and t having a nonempty common prefix and going out from i and j , respectively, then we construct the corresponding transition to the state $\left(i^{\prime },j^{\prime }\right)$ , where $i^{\prime }$ (resp. $j^{\prime }$ ) from $G_{1}$ (resp. from $G_{2}$ ) is the state that can be reached through the longest common prefix of s and t , even if both $i^{\prime }$ and $j^{\prime }$ are implicit.

We observe that, even in this case, the overall number of the transition pair checks remains the same; therefore the total size of the constructed underlying graph G remains $O\left(N_{1}m_{2}+N_{2}m_{1}\right)$ . Indeed, in the final intersection graph, all the additional nodes are at most one edge away from a previously existing node; therefore the number of additional edges outgoing from an existing node is bounded by the number of strings that can be read from that node, that is, $\min\left(m_{1},m_{2}\right)$ .

We then assign to each edge the weight w storing the length of its string label and process the nodes in reversed topological order to compute, for each node k , the value M(k) as follows: we set M(k)=0 for the nodes that do not have successors (for example, the accepting node or nodes corresponding to a pair of implicit states), and then $M\left(k\right)=\max_{k^{\prime }}\left(M\left(k^{\prime }\right)+w\left(k,k^{\prime }\right)\right)$ where $k^{\prime }$ iterates over all successors of k . By construction, for an explicit state i of $G_{1}$ and any state j of $G_{2}$ , we have M((i,j))=ℓ if and only if ℓ is equal to the maximal LCP between two strings $S_{1}$ and $S_{2}$ , where $S_{1}\in L\left(T_{1}\left[i.\;.n\right]\right)$ and $S_{2}$ is spelled starting at (explicit or implicit) state j in $G_{2}$ . Finally, for every explicit state i of $G_{1}$ we compute ${MS}_{T_{1},T_{2}}\left[i\right]=\max_{v}M\left(\left(i,v\right)\right)$ over all (explicit or implicit) states v of $G_{2}$ to obtain the output.

This ends the description of the proposed algorithm for the computation of ED Matching Statistics, which proves the following result.

Theorem 1 The ED MATCHING STATISTICS problem can be solved in $O\left(N_{1}m_{2}+N_{2}m_{1}\right)$ time by using an intersection graph of $T_{1}$ and $T_{2}$ , which can be constructed within the same complexity.

**FIGURE 4:**
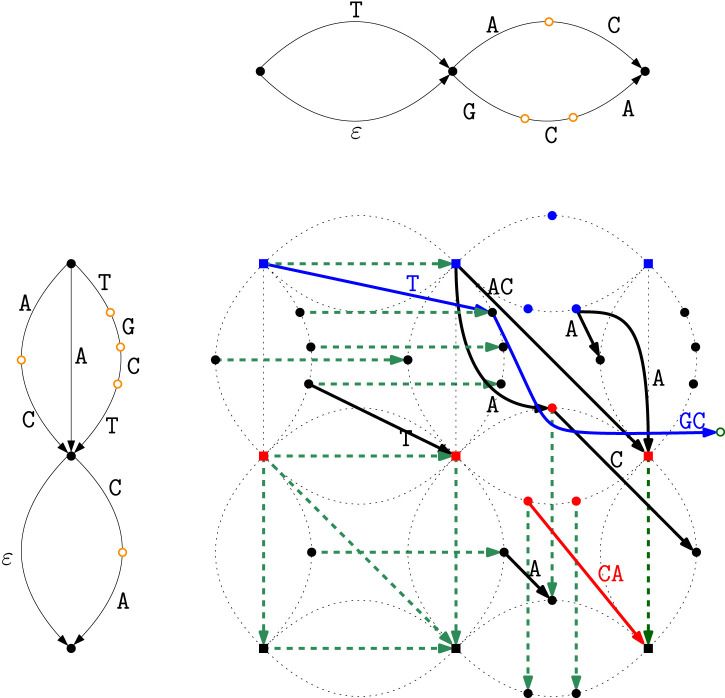
Matching Statistics of $T_{1}$ and $T_{2}$ of our running example on their intersection graph G , where, again - to simplify the understanding - we also draw $G_{1}$ and $G_{2}$ at the left and on the top, respectively. Note that this time, the pairs of implicit nodes that are reachable in a single extended transition from a pair that was previously computed are added. In the figure, there is only one such extra node, which is represented by a green open circle at the right of the graph. Here we highlight the paths that are relevant for computing the Matching Statistics array ${\text{MS}}_{T_{1},T_{2}}$ . To compute ${\text{MS}}_{T_{1},T_{2}}\left[1\right]$ , we look at the paths starting at nodes (i,j) where i is the explicit state one in the path-automaton of $T_{1}$ , and return the length of the longest label of such a path. These are the paths starting in one of the blue nodes (these are the nodes that correspond to the uppermost explicit node of $G_{1}$ paired with any node of $G_{2}$ , that is, they correspond to the uppermost dotted copy of $G_{2}$ ). The longest one of such paths (also drawn in blue) corresponds to the string TGC having length 3; therefore, ${\text{MS}}_{T_{1},T_{2}}\left[1\right]=3$ . For ${\text{MS}}_{T_{1},T_{2}}\left[2\right]$ we do the same but using as starting nodes those in red that correspond to the internal explicit node of $G_{1}$ paired with any node of $G_{2}$ (i.e., the nodes of the middle dotted copy of $G_{2}$ ). Here the longest path is drawn in red and it spells the string CA , and therefore we set ${\text{MS}}_{T_{1},T_{2}}\left[2\right]=2$ . Computing ${\text{MS}}_{T_{2},T_{1}}$ can be performed in a dual manner on the same graph, but using as starting nodes those of the leftmost dotted copy of $G_{1}$ for ${\text{MS}}_{T_{2},T_{1}}\left[1\right]$ , and those of the middle dotted copy of $G_{1}$ for ${\text{MS}}_{T_{2},T_{1}}\left[2\right]$ .

Figure 4 shows how the Matching Statistics of $T_{1}$ and $T_{2}$ of our running example can be computed on their intersection graph G . For example, to compute ${\text{MS}}_{T_{1},T_{2}}\left[1\right]$ , we look at the paths starting at nodes (i=1,j) in the path-automaton of $T_{1}$ , and return the length of the longest label of such a path. The longest one of such paths (drawn in blue) corresponds to the string TGC having length 3, and thus ${\text{MS}}_{T_{1},T_{2}}\left[1\right]=3$ .

#### 2.3.2 Breakpoint Matching Statistics

The *Breakpoint Matching Statistics* between two ED strings $T_{1}$ and $T_{2}$ refer to the notion of *breakpoint* in the genome rearrangement literature; see Baudet et al. (2010). An ED string T representing a pangenome results from a multiple sequence alignment of several genomes with the length n of T corresponding to the loci where either multiple variants or a conserved fragment begin: these are the *breakpoints* that the alignment has detected. The assumption underlying Breakpoint Matching Statistics is that biologically relevant fragments in pangenomes are those that begin at a breakpoint. The *Breakpoint Matching Statistics* between $T_{1}$ of length $n_{1}$ and $T_{2}$ of length $n_{2}$ , is an array ${\text{BMS}}_{T_{1},T_{2}}$ of length $n_{1}$ storing, for each $i\in \left[1,n_{1}\right]$ , the length of the longest local match between $T_{1}$ and $T_{2}$ that is a prefix of a string in $L\left(T_{1}\left[i.\;.n_{1}\right]\right)$ and hence starts at a breakpoint in $T_{1}$ , with the additional constraint that this must be part of a match that starts at a breakpoint in both $T_{1}$ and $T_{2}$ and ends at a breakpoint in at least one of the ED strings.

Formally, for any two ED strings, $T_{1}$ and $T_{2}$ , a *breakpoint match* (b-match) of $T_{1}$ and $T_{2}$ , for some $1\leq i_{1}\leq i_{2}\leq n_{1}$ and $1\leq j_{1}\leq j_{2}\leq n_{2}$ , is a string S such that $S\in L\left(T_{1}\left[i_{1}.\;.i_{2}\right]\right)$ and $S\in L\left(T_{2}\left[j_{1}.\;.j_{2}\right]\right)$ . Intuitively, S starts *and* ends at a breakpoint in both $T_{1}$ and $T_{2}$ .

A string S is a *left-breakpoint match* (lb-match) if (i) $S\in L\left(T_{1}\left[i_{1}.\;.i_{2}\right]\right)$ and S is a prefix of a string in $L\left(T_{2}\left[j_{1}.\;.n_{2}\right]\right)$ *or* (ii) S is a prefix of a string in $L\left(T_{1}\left[i_{1}.\;.n_{1}\right]\right)$ and $S\in L\left(T_{2}\left[j_{1}.\;.j_{2}\right]\right)$ . Intuitively, S begins at a breakpoint in both ED strings and ends at a breakpoint in at least one of the two ED strings. We denote this specific instance of S by $S_{i_{1},j_{1}}^{i_{2},j_{2}}$ . Note that any b-match is also an lb-match.

Let us now formalize the problem of computing the Breakpoint Matching Statistics that we solve in this section.

Breakpoint Matching Statistics

Input: Two ED strings, $T_{1}$ of length $n_{1}$ , cardinality $m_{1}$ and size $N_{1}$ , and $T_{2}$ of length $n_{2}$ , cardinality $m_{2}$ and size $N_{2}$ . 

Output: For each $i\in \left[1,n_{1}\right]$ , the length ${\text{BMS}}_{T_{1},T_{2}}\left[i\right]$ of a longest prefix P of a string in $L\left(T_{1}\left[i.\;.n_{1}\right]\right)$ that can be left-extended with a string from $L\left(T_{1}\left[i_{1}.\;.i-1\right]\right)$ into an lb-match $S_{i_{1},j_{1}}^{i_{2},j_{2}}$ , for some $i_{1},i_{2},j_{1},j_{2}$ .

The motivation for allowing a left-extension to an lb-match and not forcing P to be an lb-match is to maintain the property of the standard Matching Statistics of not decreasing rapidly from ${\text{MS}}_{S_{1},S_{2}}\left[i\right]$ to ${\text{MS}}_{S_{1},S_{2}}\left[i+1\right]$ .

Example 2 Let $T_{1}=\left\{\begin{array}{c} AC\\ A\\ TGCT \end{array}\right\}\cdot \left\{\begin{array}{c} \varepsilon \\ CA \end{array}\right\}$ and $T_{2}=\left\{\begin{array}{c} T\\ \varepsilon \end{array}\right\}\cdot \left\{\begin{array}{c} AC\\ GCA \end{array}\right\}$ as in Example 1. We have that ${\text{BMS}}_{T_{1},T_{2}}\left[1\right]=2$ , because P=AC starting at position i=1 is equal to S=AC , which is a b-match (for $i_{1}=i_{2}=1$ and $j_{1}=j_{2}=2$ ) and hence an lb-match. We also have ${\text{BMS}}_{T_{1},T_{2}}\left[2\right]=1$ , because P=C starting at position i=2 is left-extended to S=AC , which is an lb-match (for $i_{1}=1$ , $i_{2}=2$ and $j_{1}=j_{2}=2$ ). For both cases ( i=1 and i=2 ), we have that P is the longest possible such prefix.

We now show that the intersection graph G of $T_{1}$ and $T_{2}$ can also be used to efficiently compute their Breakpoint Matching Statistics. Indeed, the local matches that the Breakpoint Matching Statistics account for can be characterized in the intersection graph G of $T_{1}$ and $T_{2}$ as the common substrings that start at a node (i,j) where i is an explicit state of $T_{1}$ , and j can be either explicit or implicit in $T_{2}$ . Additionally, (i,j) must be reachable in G from a node $\left(i_{1},j_{1}\right)$ where $i_{1}$ and $j_{1}$ are both explicit: they correspond to a common breakpoint of the two pangenomes. Moreover, if such a substring ends at node $\left(i_{2},j_{2}\right)$ , then at least one state among $i_{2}$ and $j_{2}$ must be explicit. Notice that, should j be an explicit node, then the reachability condition above can be fulfilled by $j=j_{1}$ itself; in that case we also have $i=i_{1}$ . On the other hand, if j is implicit, then it must be that $i_{1}\neq i$ and $j_{1}\neq j$ .

**FIGURE 5:**
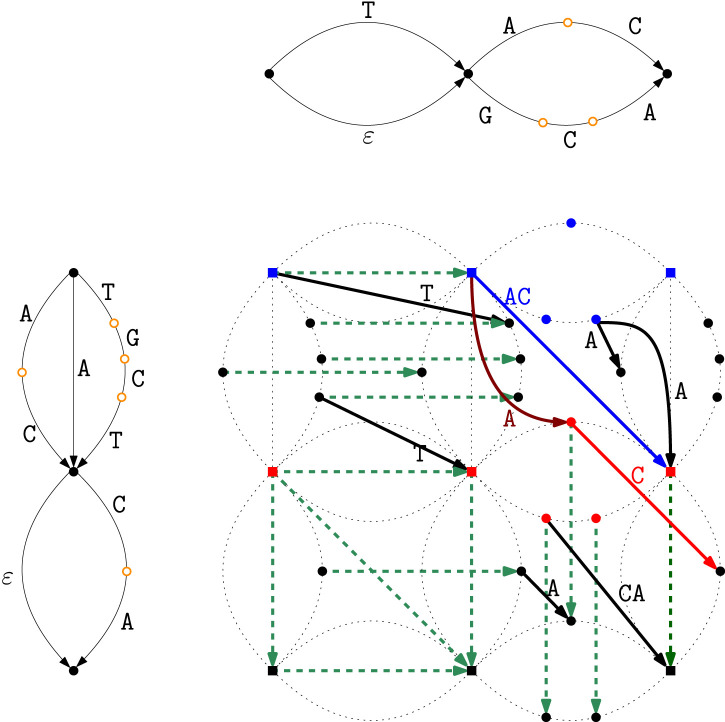
Breakpoint Matching Statistics computation in the intersection graph G of $T_{1}$ and $T_{2}$ . To compute ${\text{BMS}}_{T_{1},T_{2}}\left[1\right]$ , the candidate starting nodes of the match in G are those in blue: nodes (i,j) where i is an explicit state of $T_{1}$ in the uppermost dotted copy of $G_{2}$ , and j is either an explicit state of $T_{2}$ (squared blue nodes) or an implicit one (circled blue nodes). Note that TGC is the longest match that starts at the first set of $T_{1}$ but it does not fulfill the conditions for the Breakpoint Matching Statistics because it does not end at any breakpoint; for the same reason, TG is also not a good candidate match. The occurrence of AC corresponding to the blue edge starts at a blue square node; hence it is reachable from the node itself that corresponds to a pair of explicit states, and it ends at a node that is again a pair of explicit states, and hence a breakpoint for both $T_{1}$ and $T_{2}$ . There is no longer match satisfying these conditions; therefore we set ${\text{BMS}}_{T_{1},T_{2}}\left[1\right]=2$ . For ${\text{BMS}}_{T_{1},T_{2}}\left[2\right]$ we do the same but use as starting nodes those in red that correspond to the internal explicit node of $G_{1}$ paired with any node of $G_{2}$ (i.e., the nodes of the middle dotted copy of $G_{2}$ ). The red path spelling C : (i) is a prefix in $T_{1}\left[2\right]$ starting at an explicit node of $T_{1}$ ; (ii) is reachable from a square node in G by spelling A in both strings (curved brown red edge labeled with A ); and (iii) ends where $T_{2}\left[2\right]$ does, that is, at a breakpoint. Since this is the longest such path in G , we set ${\text{BMS}}_{T_{1},T_{2}}\left[2\right]=1$ . Note, for example, that the match CA that occurs in $T_{1}\left[2\right]$ and inside $T_{2}\left[2\right]$ cannot be used for ${\text{BMS}}_{T_{1},T_{2}}\left[2\right]$ because it starts at a node that is not reachable from a pair of explicit nodes, meaning that it is not upperbounded by a breakpoint in $T_{2}$ . Computing ${\text{BMS}}_{T_{2},T_{1}}$ , which is of size $n_{2}=2$ , can be done in a dual manner on the very same graph, using as starting nodes those of the leftmost dotted copy of $G_{1}$ for ${\text{BMS}}_{T_{2},T_{1}}\left[1\right]=2$ (obtained by traversing an ε -transition and then AC ), and those of the middle dotted copy of $G_{1}$ for ${\text{BMS}}_{T_{2},T_{1}}\left[2\right]=2$ ( AC again).

Figure 5 shows the computation of the Breakpoint Matching Statistics in the intersection graph G of our running example. For example, for ${\text{BMS}}_{T_{1},T_{2}}\left[1\right]$ , we use the occurrence of AC (blue edge) starting at a node that corresponds to a pair of explicit states and ending at a node that also corresponds to a pair of explicit states (a breakpoint for both $T_{1}$ and $T_{2}$ ). No longer match satisfies these conditions; hence, we set ${\text{BMS}}_{T_{1},T_{2}}\left[1\right]=2$ .

Notice that the Breakpoint Matching Statistics require more restricted matches tī ED Matching Statistics. Indeed we have that for any two ED strings $T_{1}$ and $T_{2}$ , it holds that ${\text{BMS}}_{T_{1},T_{2}}\left[i\right]\leq {\text{MS}}_{T_{1},T_{2}}\left[i\right]$ for all $1\leq i\leq n_{1}$ . It should be clear that Breakpoint Matching Statistics can be computed within the same complexities as the ones described in Theorem 1. We thus obtain the following.

Theorem 2 The Breakpoint Matching Statistics problem can be solved in $O\left(N_{1}m_{2}+N_{2}m_{1}\right)$ time by using an intersection graph of $T_{1}$ and $T_{2}$ , which can be constructed within the same complexity.

### 2.4 Our measures for comparing pangenomes

In this section we describe a similarity and a distance measure for pangenome comparison. These measures can be derived from either the MS or the BMS array. We assume that these have been pre-computed.

We consider both arrays ${\text{MS}}_{T_{1},T_{2}}$ and ${\text{MS}}_{T_{2},T_{1}}$ (resp. ${\text{BMS}}_{T_{1},T_{2}}$ and ${\text{BMS}}_{T_{2},T_{1}}$ ) as the Matching Statistics is not *per se* a symmetric notion: the two arrays do not even need to have the same size (when $n_{1}\neq n_{2}$ ). A simple solution for a similarity measure is to consider the sum of the two averages: each array is turned into a number being the average of its values, and the sum makes everything symmetric. Thus, we define the *similarity measure* $MS\left(T_{1},T_{2}\right)$ (resp. $BMS\left(T_{1},T_{2}\right)$ ) between $T_{1}$ and $T_{2}$ as follows:

$$MS\left(T_{1},T_{2}\right)=MS\left(T_{2},T_{1}\right)=\frac{\sum_{i\in \left[1,n_{1}\right]}{\text{MS}}_{T_{1},T_{2}}\left[i\right]}{n_{1}}+\frac{\sum_{j\in \left[1,n_{2}\right]}{\text{MS}}_{T_{2},T_{1}}\left[j\right]}{n_{2}}$$

and

$$BMS\left(T_{1},T_{2}\right)=BMS\left(T_{2},T_{1}\right)=\frac{\sum_{i\in \left[1,n_{1}\right]}{\text{BMS}}_{T_{1},T_{2}}\left[i\right]}{n_{1}}+\frac{\sum_{j\in \left[1,n_{2}\right]}{\text{BMS}}_{T_{2},T_{1}}\left[j\right]}{n_{2}}$$

We now move further in order to design a notion of *distance* between pangenomes based on $MS\left(T_{1},T_{2}\right)$ (resp. $BMS\left(T_{1},T_{2}\right)$ ). Unlike the notion of similarity, the distance has to decrease when the two pangenomes get more similar; hence, following a standard procedure, we invert the similarity measure while normalizing over the logarithm of the size of the pangenome. The reason for the normalization is that the values inside arrays ${\text{MS}}_{T_{1},T_{2}}$ and ${\text{BMS}}_{T_{1},T_{2}}$ are affected by the sizes of both strings–a very short ED string cannot contain a long match. Therefore, for a given $T_{1}$ , to account for the size $N_{2}$ of $T_{2}$ , we normalize $MS\left(T_{1},T_{2}\right)$ (resp. $BMS\left(T_{1},T_{2}\right)$ ) by $\log N_{2}$ and then invert^4^, thus obtaining $\log N_{2}/MS\left(T_{1},T_{2}\right)$ (resp. $\log N_{2}/BMS\left(T_{1},T_{2}\right)$ ). Again, this gives rise to a non-symmetric notion, while symmetry is a desired property for a distance. Another desired property is reflexivity, requiring any pangenome to have distance zero from itself. The latter can be ensured by subtracting^5^ a “correction term” as follows:

$$\bar{d}\left(T_{1},T_{2}\right)=\frac{\log N_{2}}{MS\left(T_{1},T_{2}\right)}-\frac{\log N_{1}}{MS\left(T_{1},T_{1}\right)}.$$

and

$$\bar{bd}\left(T_{1},T_{2}\right)=\frac{\log N_{2}}{BMS\left(T_{1},T_{2}\right)}-\frac{\log N_{1}}{BMS\left(T_{1},T_{1}\right)}.$$

thus guaranteeing that $\bar{d}\left(T_{1},T_{1}\right)=\bar{bd}\left(T_{1},T_{1}\right)=0$ for any nonempty $T_{1}$ . However, both $\bar{d}$ and $\bar{bd}$ are not symmetric yet, and hence, we finally ensure that our distance is *symmetric* resorting again to the sum. Therefore, we set:

$$d\left(T_{1},T_{2}\right)=d\left(T_{2},T_{1}\right)=\bar{d}\left(T_{1},T_{2}\right)+\bar{d}\left(T_{2},T_{1}\right).$$

and

$$bd\left(T_{1},T_{2}\right)=bd\left(T_{2},T_{1}\right)=\bar{bd}\left(T_{1},T_{2}\right)+\bar{bd}\left(T_{2},T_{1}\right).$$

Our d and bd distances resemble an analogous widely used distance measure for standard sequences such as *MissMax* (Pizzi, 2016) that is based on Matching Statistics with mismatches, and *kmacs* (Ulitsky et al., 2006; Leimeister and Morgenstern, 2014; Thankachan et al., 2017) that is based on an approximation of Matching Statistics with mismatches.

## 3 Experiments

We implemented the $O\left(N_{1}m_{2}+N_{2}m_{1}\right)$ -time algorithm for solving EDSI as well as an $O\left(N_{1}N_{2}\right)$ -time algorithm for EDSI based on the classic product automaton construction. We also implemented the $O\left(N_{1}m_{2}+N_{2}m_{1}\right)$ -time algorithm computing both Matching Statistics notions as well as the downstream similarities and distance measures for any two ED strings. All implementations were written in C++ and the source code is freely available at https://github.com/urbanslug/junctions. We compiled all implementations with gcc version 12.2.0 at optimization level (-O3).

### 3.1 Efficiency on simulated data

In this section, we compare the running time of our EDSI with that of the naïve method and with the parameters that define the characteristics of the input ED strings, with the purpose of validating the time efficiency of our algorithm and show how it actually improves in practice with respect to the baseline quadratic running time. In order to do that, we use synthetic data generated on the alphabet {A,C,G,T} .

The synthetic ED strings were generated using another tool of ours named SimED (https://github.com/urbanslug/simed). The tool starts by generating a random standard string of length W over the DNA alphabet, assuming a uniform distribution of letters. This is considered to be an initial sequence. We can view this as an ED string with N=W and n=m=1 . The SimED tool assumes a very simple evolutionary model (where each position has an equal chance of mutating, and each letter has the same probability of occurring at any position) and generates an ED string from the initial sequence based on the following input parameters.

• W as the length of the initial random (not ED yet) string;

• d as the number of positions where a set of multiple strings occurs, given as a percentage of W (that is, d is the fraction of degenerate positions);

• S as the maximum number of strings in any set of the resulting ED string;

• L as the maximum length of the strings in any set of the resulting ED string.

As aforementioned, the tool first generates a standard string uniformly at random, which we denote by X (|X|=W) . It then samples δ=⌈dW⌉ non-overlapping length- L substrings of X uniformly at random. We denote these by $X\left[i_{1}.\;.i_{1}+L-1\right],\ldots ,X\left[i_{\delta }.\;.i_{\delta }+L-1\right]$ , where $i_{j}+L\leq i_{j+1}$ for j∈[1,δ−1] . For every j∈[1,δ] , it picks a uniformly random integer s from [1,S] and produces s−1 strings of uniformly random lengths from [0,L] , each string generated uniformly at random; these s−1 strings and the original fragment $X\left[i_{j}.\;.i_{j}+L-1\right]$ form a set $D_{i_{j}}$ of strings. Finally it sets T as $X\left[1.\;.i_{1}-1\right]\cdot D_{i_{1}}\cdot X\left[i_{1}+L.\;.i_{2}-1\right]\cdot D_{i_{2}}\cdots D_{i_{\delta }}\cdot X\left[i_{\delta }+L.\;.W\right]$ . Note that T is indeed an ED string; we denote its length, cardinality, and size by n,m,N , respectively. If we choose d,S,W such that (δ+δ⋅S)≪W then we have that m≤(δ+δ⋅S)≪W≤N⇒m≪N . It is worth noting that if the same initial string X is used to generate two distinct ED strings, then X will appear in their (nonempty) intersection.

Starting from the same base sequence X of length W , in each experiment described in this section, we used the same parameters d,L and S to generate both $T_{1}$ and $T_{2}$ . Thus, X guarantees a nonempty intersection between $T_{1}$ and $T_{2}$ , and both implementations always answered YES (as expected) without a premature ending (hence, detecting their worst-case running time). As expected, the improved $O\left(N_{1}m_{2}+N_{2}m_{1}\right)$ -time implementation of EDSI was faster than the naïve $O\left(N_{1}N_{2}\right)$ -time implementation in all datasets, especially with longer variants and/or with short widely interspersed variants, that is for ED strings where m≪N . Results are shown below.

We report a table for each set of parameters, and in each table, we show the data for different starting synthetic string lengths W , up to |W|=100k bases. The data reported in the columns of Tables 1, 2 are: the length W of the initial string, the size $N_{1}$ and cardinality $m_{1}$ of the first synthetic ED string, the size $N_{2}$ and cardinality $m_{2}$ of the second synthetic ED string, and the time taken by the Naïve method and by EDSI, both measured in seconds. The parameters d (frequency of positions with multiple variants), S (maximum number of variants in such positions), and L (maximum length of such variants) determine the degree of *degeneracy* of the ED strings. As shown below, we have m≪N because (i) wherever the sequence is not degenerate, N grows linearly with W while m is constant, and (ii) wherever there is a degenerate position, N∈O(S×L) while m∈O(S) . This explains why our $O\left(N_{1}m_{2}+N_{2}m_{1}\right)$ -time algorithm is much faster than the $O\left(N_{1}N_{2}\right)$ -time one.

**TABLE 1:** Results with simulation parameters: d=0.1% with S=3 , L=3 and with S=5 , L=5 .

W	$N_{1}$	$m_{1}$	$N_{2}$	$m_{2}$	Naïve (s)	EDSI (s)
S=3 and L=3						
10k	10,019	36	10,023	38	0.69	0.04
30k	30,062	107	30,071	107	6.20	0.14
50k	50,106	173	50,110	172	17.57	0.29
100k	100,225	354	100,203	344	72.81	0.47
S=5 and L=5						
10k	10,084	49	10,066	50	0.68	0.06
30k	30,198	144	30,212	148	6.21	0.16
50k	50,381	244	50,358	250	18.00	0.29
100k	100,837	515	100,776	500	74.04	0.65

**TABLE 2:** Simulation parameters: d=1% with S=3 , L=3 , with S=5 , L=5 , and with S=5 , L=10 .

W	$N_{1}$	$m_{1}$	$N_{2}$	$m_{2}$	Naïve (s)	EDSI (s)
S=3 and L=3						
10k	10,218	346	10,232	348	0.70	0.05
30k	30,688	1,064	30,659	1,040	6.46	0.21
50k	51,155	1758	51,104	1752	18.84	0.48
100k	102,227	3,469	102,258	3,497	77.86	1.71
S=5 and L=5						
10k	10,838	504	10,796	494	0.80	0.06
30k	32,362	1,479	32,415	1,505	7.36	0.25
50k	54,098	2,508	54,146	2,525	20.84	0.56
100k	108,071	4,987	107,947	4,986	84.62	1.89
S=5 and L=10						
10k	11,696	498	11,803	500	0.96	0.06
30k	35,405	1,531	35,140	1,495	8.83	0.25
50k	58,745	2,503	58,659	2,484	25.22	0.59
100k	117,444	4,985	117,417	4,989	101.10	1.97

The tables show that the Naïve scales quadratically in the size of the ED strings while EDSI is much faster as m≪N . A comparison of the second and third experiments reported in Tables 2 highlights how, when only L grows (it doubles from 5 to 10 while d and S remain 1% and 5, respectively), our tool has basically the same performance whereas the Naïve becomes slower. The explanation is that when L grows, only N grows while m does not (as we can see), and hence m and N diverge even more. Finally, we remark that the parameter that most affects m/N (i.e., the ratio of our asymptotic gain with respect to the Naïve) is d , as the comparison of Tables 1 and 2 shows for the corresponding values of S and L .

These experiments were conducted on a laptop with a 64 bit 11th Gen Intel(R) Core(TM) i7-11800H 8 core processor and 16 GB of RAM. Timings were recorded using std::chrono from the C++ standard library.

### 3.2 Efficiency on human genome data

In this section, we present some experiments for the running time of EDSI on real human genome data with variants. The goal is to show that our tool is fast even on real data, as the ratio between m and N is not too large for real pangenomes built out of real human genome fragments and their Variant Call Format (VCF) data. We built ED strings for human genome data from the GRCh38.p13 dataset, specifically from HLA-B in chromosome VI as well as the actin beta (ACTB) gene in chromosome VII. We used human genomic sequence data in the FASTA format and variation data in the Variant Call Format (.vcf file) from the following three databases: 1000G https://www.internationalgenome.org/ (2024), TOPmed https://topmed.nhlbi.nih.gov/ (2024), and gnomAD https://gnomad.broadinstitute.org/ (2024).

The human leukocyte antigen (HLA) gene is contained in the major histocompatibility complex on the p arm (chromosomal region 6p21.33) of Chromosome VI which is known to be one of the most polymorphic regions of the human genome. The HLA gene is involved in immune system regulation (Crux and Elahi, 2017; Romero-Sánchez et al., 2021) and is found in the region between positions 31,353,872 and 31,367,067 (hence it is 13 kb long). ACTB is a highly conserved gene in humans that codes for several proteins involved in cell structure and integrity. For each genome fragment (HLA and ACTB data), and for each database (out of the three 1000G, TOPmed, and gnomAD), we selected from the .vcf file only the variants that are recorded in that specific database, and we updated the regions containing variation, as denoted in the.vcf file into sets containing both the original in the reference and the variants contained in.vcf, thus creating an ED string. We performed this in two ways: one for all variants of that database for that genome fragment, and one by selecting the SNPs variants only. We then used AEDSO (https://github.com/urbanslug/aedso) to build the ED strings. Data download links and commands used are available at https://github.com/urbanslug/junctions/blob/master/test_data/human.org.

In summary, we have two ED strings (one with all variants and one with SNPs only) per each database, and each genome fragment. We remark that all of these ED strings include the original non-mutated string in the language; hence for each pair *the intersection is nonempty*. This ensures detecting a running time of EDSI without a premature ending due to empty intersection: we ran EDSI for all pairs. For the HLA data, Table 3 shows the sizes (and types) of the ED strings and the running times of a few of these experiments. Table 4 shows results for the ACTB data. We observe that EDSI improves over Naïve by one order of magnitude whenever m≪N (1000G and gNomad variants datasets), and still improves over the Naïve even when N/m is a small constant, like with TOPMed data.

**TABLE 3:** ED strings with databases and VCF and sizes, and time (in seconds) required by Naïve and by EDSI for HLA data.

${\text{DB}}_{1}$	${\text{VCF}}_{1}$	$N_{1}$	$m_{1}$	${\text{DB}}_{2}$	${\text{VCF}}_{2}$	$N_{2}$	$m_{2}$	Naïve (s)	EDSI (s)
1000G	all	13,332	224	1000G	SNP	13,247	161	1.25	0.05
TOPMed	all	15,090	3,452	gNomad	SNP	13,941	1785	2.11	1.06
gNomad	all	14,436	2044	TOPMed	SNP	14,355	3,170	2.10	1.13

**TABLE 4:** ED strings with databases and VCF and sizes, and time (in seconds) required by Naïve and by EDSI for ACTB data.

${\text{DB}}_{1}$	${\text{VCF}}_{1}$	$N_{1}$	$m_{1}$	${\text{DB}}_{2}$	${\text{VCF}}_{2}$	$N_{2}$	$m_{2}$	Naïve (s)	EDSI (s)
1000G	all	37,019	644	gNomad	SNP	37,876	3,146	9.82	0.53

Finally, to conduct an experiment on these data with larger inputs, we picked a larger fragment of reference from Chromosome VI (spanning over the HLA region) of length 100Kb, and we repeated the same procedure as above. Table 5 presents the results of the experiment. We observe that even for these longer ED strings, EDSI is generally significantly faster than the Naïve method, especially on data such as that of the 1000G variants dataset–therein the ratio between N and m is larger than in the other data.

**TABLE 5:** ED strings with databases and VCF and sizes, and time (in seconds) required by Naïve and by EDSI for a fragment of data.

${\text{DB}}_{1}$	${\text{VCF}}_{1}$	$N_{1}$	$m_{1}$	${\text{DB}}_{2}$	${\text{VCF}}_{2}$	$N_{2}$	$m_{2}$	Naïve (s)	EDSI (s)
1000G	all	100,951	3,730	1000G	SNP	101,252	2,753	73	1.12
TOPMed	all	113,111	21,253	TOPMed	SNP	108,669	18,931	99.04	21.44
gNomad	all	130,918	42,572	gNomad	SNP	117,793	38,877	150.72	109.83

These experiments were conducted on a laptop with a 64 bit 11th Gen Intel(R) Core(TM) i7-11800H 8 core processor and 16 GB of RAM. Timings were recorded using std::chrono from the C++ standard library.

### 3.3 Similarity of SARS-CoV-2 clades

To demonstrate the effectiveness of the Breakpoint Matching Statistics and the similarity measure based on them, we computed the BMS arrays and the BMS similarity measures for all pairs of clades of real SARS-CoV-2 data downloaded from NextStrain^6^ Hadfield et al. (2018), a platform collecting SARS-CoV-2 evolution analyses, built out of GenBank https://www.ncbi.nlm.nih.gov/genbank/ (2024) data. As an example of the NextStrain report, Figure 7 shows a phylogenetic tree of 3357 SARS-CoV-2 genomes sampled between December 2019 and August 2023, constructed using a complex pipeline described in https://docs.nextstrain.org/en/latest/learn/parts.html (2024).

We selected 35 SARS-CoV-2 clades as classified by NextStrain in https://nextstrain.org/ncov/open/global/alltime (2024) and, within each of them, we downloaded randomly selected individual genome samples (10 when available, and less otherwise), ruling out a few clades with too few samples: we were left with 31 clades. We provide the raw datasets at https://github.com/urbanslug/junctions/tree/master/test/_data/SARS/_CoV/_2.

For each clade, we constructed a multiple sequence alignment (MSA) using abPOA (Gao et al., 2020) and, from each such MSA, we generated the corresponding ED string using msa2eds ^7^. Our tool msa2eds constructs ED strings from an MSA by simply collapsing 100% conserved columns (that is, columns with the same letter in all variants) into solid letters in the ED string, and into sets of distinct variants otherwise.

For all pairs h,k of these 31 SARS-CoV-2 clades, we computed the Breakpoint Matching Statistics arrays ${\text{BMS}}_{T_{k},T_{h}}$ and ${\text{BMS}}_{T_{h},T_{k}}$ , and the consequent pairwise similarity $BMS\left(T_{k},T_{h}\right)$ ^8^.

**FIGURE 6:**
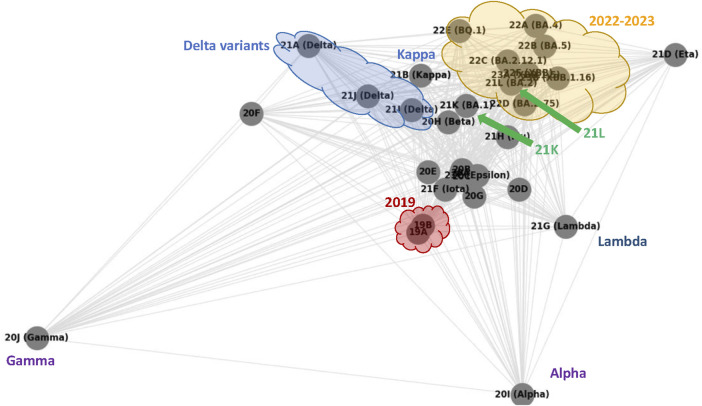
SARS-CoV-2 clades pairwise similarity graph generated according to average Breakpoint Matching Statistics. The annotation (all non grey nor black graphics and text) highlights similarities with Figure 7.

Figure 6 shows the graph generated using NetworkX’s *spring_layout* toolkit (Hagberg et al., 2008) when given all pairwise BMS among the 31 clades as input parameters. NetworkX’s algorithm simulates a force-directed representation of the network, treating nodes as repelling objects and edges as springs that hold the nodes close according to the input similarity measures. The simulation continues until the positions are close to an equilibrium, which results in a graph in which closely-related (that is, similar according to our BMS measure) clades are clustered together. We also computed, for all pairs h,k of the clades, the Matching Statistics arrays ${\text{MS}}_{T_{k},T_{h}}$ and ${\text{MS}}_{T_{h},T_{k}}$ , and the consequent pairwise similarity $MS\left(T_{k},T_{h}\right)$ . However, the graph built using NetworkX’s *spring_layout* with the Matching Statistics similarity measures (data not shown here) did not exhibit clade clusters as significant as those in Figure 6 generated with the Breakpoint Matching Statistics similarity.

**FIGURE 7:**
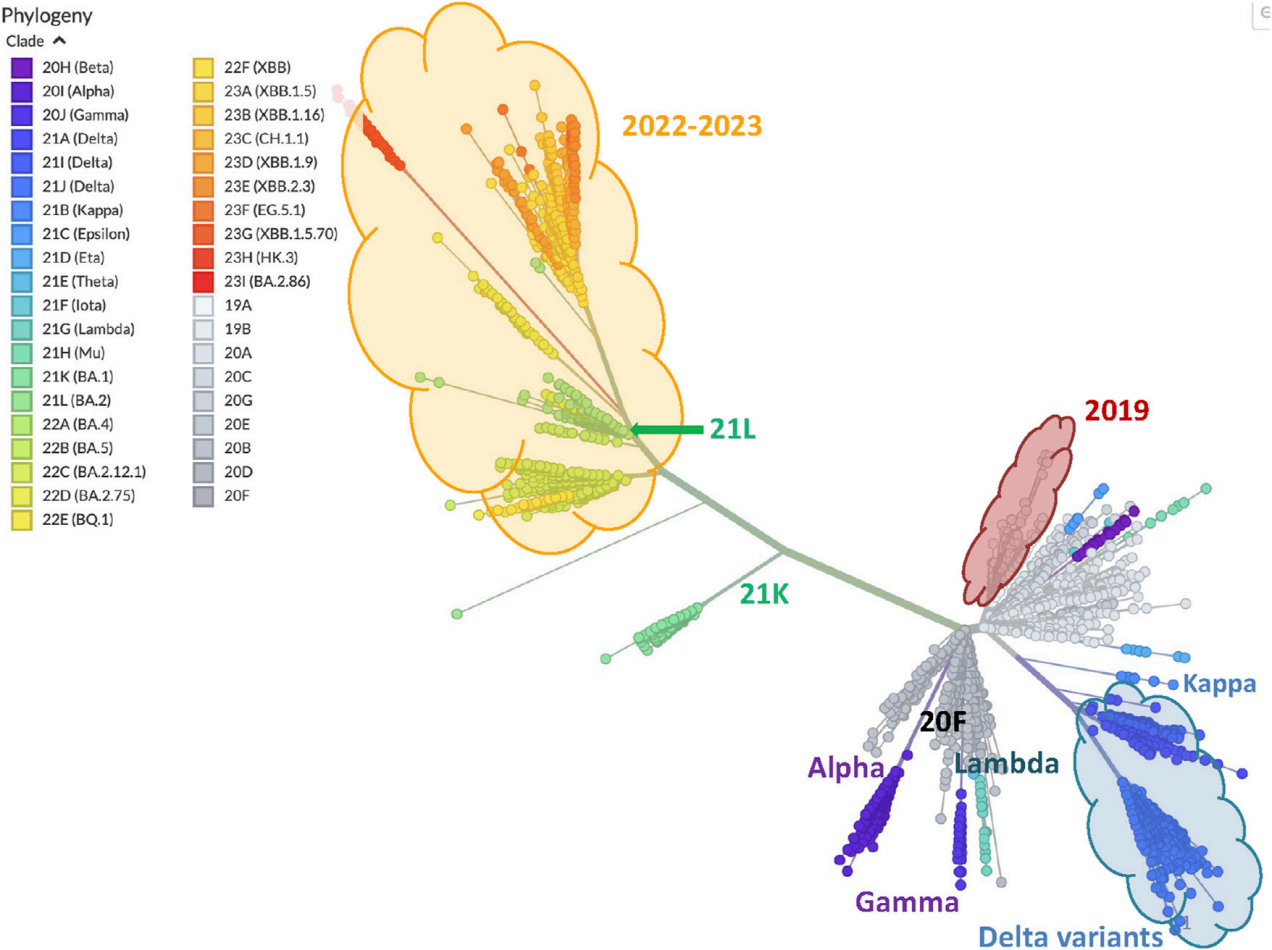
Phylogeny of 3357 SARS-CoV-2 genomes samples. The figure is generated and downloaded from Nextstrain https://nextstrain.org/ncov/open/global/all-time (2024), and some annotation is added here to highlight similarities with the graph of Figure 6.

As our annotation shows, the graph in Figure 6 sketches a phylogenetic network of SARS-CoV-2 clades that essentially reproduces the NextStrain phylogeny shown in Figure 7. The former is a complete graph in which all edges are present, and their length is related to our similarity measure (the closer, the more similar); the latter is a typical unrooted phylogenetic tree structure in which clades similarities group subpopulations into subtrees (the closer the common ancestor is, the more similar).

• **2019 clades**. The two 2019 clades 19A and 19B, are very close in Figure 6 (circled by a red cloud shape), and belong to the same subtree in Figure 7 of the NextStrain phylogeny.

• **Delta and Kappa variant clades**. The three clades 21A, 21I and 21J, which all belong to the *Delta variant* are clustered together and highlighted by a (blue) cloud shape at the top of Figure 6 as they turn out to have a higher similarity compared to each other. The grouping of these clades reproduces that of the NextStrain phylogeny shown in Figure 7, where the three Delta clades are the cluster of blue branches at the bottom right. Moreover, in both figures clade 21B of the *Kappa variant* is very close to the Delta variants.

• **20F, Gamma and Lambda variant clades**. In the graph in Figure 6, the 20F clade and the *Lambda* and *Gamma variants* seem to be outliers, as they stand slightly away from everything else. Indeed, by looking at the data and maps in NextStrain^6^, it turns out that variant gamma is, in fact, peculiar, as it has lasted over a year with a quite regular but limited incidence, and checking its location on the world map of NextStrain^6^, we can actually see that it was diffused almost exclusively in South America, thus explaining its isolated position in our graph. Similarly, the *Lambda variant* has only been observed in western South America. Finally, clade 20F turns out to have been observed basically only in center Australia. These type of isolated clades are also highlighted as independent of each other in Figure 7, where subtrees that include their sample are all rooted in the main thick branch of the phylogeny (as it is better visible in NextStrain^6^ than in Figure 7).

• **Alpha variant clade**. The *alpha variant* of SARS-CoV-2 has spread worldwide, with a high incidence for over 2 years. In Figure 7 its many samples all lay in a subtree rooted (like those of the *Gamma* and *Lambda variants*) directly at the main branch of the phylogeny; accordingly, in the graph of Figure 6, the node corresponding to the *Alpha variant* pangenome is not specifically close to any other clade.

• **2020 clades**. Apart from the aforementioned variants, not surprisingly, all the other 2020 clades appear in Figure 6 and are all clustered in the center of the graph.

• **2022 and 2023 clades**. Finally, the late variants of 2023 and their 2022 ancestors are all clustered at the top right of our graph, again highlighted by a cloud (yellow) shape. This is again in agreement with the NextStrain phylogeny, as we observe these clades at the top of Figure 7. In both figures, we also observe that two 2021 clades appear as outliers inside (21L) or close to (21K) the areas of these late variants (they are pointed by green arrows in both figures).

Thus, we can conclude that our BMS similarity measure allowed us to reproduce the clade clustering of an established phylogeny for SARS-CoV-2 data. For the computing performance, for each pair of clades, we recorded the CPU time required to build the two ED strings out of the MSAs, compute their intersection graph, and compute the BMS array and its average. The 465 pairwise similarity computations required 17 h of CPU time in total and 2 min on average (30 min for the slowest pair); we remark that these computations can be performed in parallel. Memory usage ranged from 22MB to 361 MB. These values show the moderate resource requirements of our methods.^9^ These experiments were run on a DELL PowerEdge R750 machine, used in non-exclusive mode, with 2 Intel(R) Xeon(R) Gold 5318Y CPUs, each running at 2.10 GHz and using 24 physical cores (and 48 hyperthreading cores). The main memory is shared and is of size 992 GB. The operating system used is Ubuntu 22.04.2 LTS.

## 4 Future work

We plan to investigate methods for local comparison of ED strings, that is, devising efficient methods to find fragments that are common to two or more ED strings (like the fragments we detect with Matching Statistics) but that are not necessarily identical in all of their occurrences (unlike those we detect with Matching Statistics). This could be achieved by means of a preliminary preprocessing filtering step such as those of Peterlongo et al. (2009) for edit distance and Peterlongo et al. (2005), (2008) for Hamming distance. This filtering step could possibly be paired with suitable notions of maximality in frequency (Federico and Pisanti, 2009) or in conservation (Grossi et al., 2009; 2011) for the common fragments in order to avoid redundancy in the output results.

## Data Availability

The original contributions presented in the study are included in the article/supplementary material, further inquiries can be directed to the corresponding author.

## Footnotes

1 A preliminary step on degenerate strings (D strings), that is, a restricted version of ED strings, was made by Alzamel et al. (2018), (2020).

2 A *breakpoint* in a genome is a location on a chromosome where DNA might get deleted, inverted, or swapped around Sankoff and Blanchette (1998).

3 This can be done using the folklore product automaton construction (Lawson, 2004).

4 We assume that $MS\left(T_{1},T_{2}\right),BMS\left(T_{1},T_{2}\right)>0$ .

5 For a nonempty $T_{1}$ , the quantities $MS\left(T_{1},T_{1}\right)$ and $BMS\left(T_{1},T_{1}\right)$ are always greater than zero

6
https://nextstrain.org/ncov/open/global/all-time

7
https://github.com/urbanslug/junctions

8 A complete table with all pairwise BMS scores among clades is available at the following url:https://github.com/urbanslug/junctions/blob/master/test_data/SARS/CoV/2/BPMS/similarity.tsv.

9 Precise measurements are listed here: https://github.com/urbanslug/junctions/blob/master/test_data/covid.org.
